# Cadmium Exposure and All-Cause and Cardiovascular Mortality in the U.S. General Population

**DOI:** 10.1289/ehp.1104352

**Published:** 2012-04-02

**Authors:** Maria Tellez-Plaza, Ana Navas-Acien, Andy Menke, Ciprian M. Crainiceanu, Roberto Pastor-Barriuso, Eliseo Guallar

**Affiliations:** 1 Department of Epidemiology, Johns Hopkins Bloomberg School of Public Health, Baltimore, Maryland, USA; 2 Department of Epidemiology, Atherothrombosis and Imaging, National Center for Cardiovascular Research (CNIC), Madrid, Spain; 3 Department of Environmental Health Sciences, Johns Hopkins Bloomberg School of Public Health, Baltimore, Maryland, USA; 4 Welch Center for Prevention, Epidemiology and Clinical Research, Johns Hopkins Medical Institutions, Baltimore, Maryland, USA; 5 Department of Biostatistics, Johns Hopkins Bloomberg School of Public Health, Baltimore, Maryland, USA; 6 National Center for Epidemiology, Instituto de Salud Carlos III, Madrid, Spain; 7 CIBER en Epidemiología y Salud Publica (CIBERESP), Madrid, Spain; 8 Department of Medicine, Johns Hopkins Medical Institutions, Baltimore, Maryland, USA

**Keywords:** cadmium, cardiovascular disease, mortality, NHANES, survey

## Abstract

Background: Urine cadmium concentrations were associated with all-cause and cardiovascular mortality in men in the 1988–1994 U.S. National Health and Nutrition Examination Survey (NHANES) population. Since 1988, cadmium exposure has decreased substantially in the United States. The associations between blood and urine cadmium and cardiovascular disease (CVD) mortality at more recent levels of exposure are unknown.

Objectives: We evaluated the prospective association of blood and urine cadmium concentrations with all-cause and CVD mortality in the 1999–2004 U.S. population.

Methods: We followed 8,989 participants who were ≥ 20 years of age for an average of 4.8 years. Hazard ratios for mortality end points comparing the 80th to the 20th percentiles of cadmium distributions were estimated using Cox regression.

Results: The multivariable adjusted hazard ratios [95% confidence intervals (CIs)] for blood and urine cadmium were 1.50 (95% CI: 1.07, 2.10) and 1.52 (95% CI: 1.00, 2.29), respectively, for all-cause mortality, 1.69 (95% CI: 1.03, 2.77) and 1.74 (95% CI: 1.07, 2.83) for CVD mortality, 1.98 (95% CI: 1.11, 3.54) and 2.53 (95% CI: 1.54, 4.16) for heart disease mortality, and 1.73 (95% CI: 0.88, 3.40) and 2.09 (95% CI: 1.06, 4.13) for coronary heart disease mortality. The population attributable risks associated with the 80th percentile of the blood (0.80 μg/L) and urine (0.57 μg/g) cadmium distributions were 7.0 and 8.8%, respectively, for all-cause mortality and 7.5 and 9.2%, respectively, for CVD mortality

Conclusions: We found strongly suggestive evidence that cadmium, at substantially low levels of exposure, remains an important determinant of all-cause and CVD mortality in a representative sample of U.S. adults. Efforts to further reduce cadmium exposure in the population could contribute to a substantial decrease in CVD disease burden.

Cadmium is a toxic metal present in certain foods, in tobacco smoke, and in ambient air [Agency for Toxic Substances and Disease Registry (ATSDR) 2008; Nordberg et al. 2007]. In the Third National Health and Nutrition Examination Survey (NHANES III, 1988–1994), urine cadmium levels were associated with all-cause mortality and CVD mortality in men, both overall and in men who had never smoked (Menke et al. 2009). Although exposure levels have decreased in recent decades (Tellez-Plaza et al. 2012), cadmium levels are still associated with prevalent CVD, bone and kidney disease, and self-reported breast cancer in NHANES data collected after 1999 (Egwuogu et al. 2009; Gallagher et al. 2008, 2010; Navas-Acien et al. 2004, 2005, 2009; Peters et al. 2010; Tellez-Plaza et al. 2008, 2010). However, the prospective association between blood and urine cadmium concentrations with mortality at recent levels of exposure in NHANES has not been investigated.

In the present study, we evaluated the prospective association of cadmium exposures with all-cause and CVD mortality in NHANES participants who were recruited in 1999–2004 and followed through 2006. In NHANES 1999–2004, cadmium was measured in whole blood and in urine. Both biomarkers reflect cumulative exposure, although blood cadmium also reflects short-term fluctuations in cadmium exposure (ATSDR 2008; Järup et al. 1983; Nordberg et al. 2007). We hypothesized that urine and blood cadmium levels would be associated with increased all-cause and CVD mortality, with an interest in evaluating potential effect modification by sex and smoking status.

## Methods

*Study population.* NHANES uses a complex multistage sampling design to obtain representative samples of the noninstitutionalized U.S. population [National Center for Health Statistics (NCHS) 2008]. For this analysis, we used data from 14,213 adults ≥ 20 years of age who participated in the NHANES 1999–2004 interviews and examinations. The overall participation rate was 70%. We excluded 772 pregnant women, 661 participants who were missing blood cadmium measures, 4 participants with urine cadmium corrected for molybdenum interference equal to zero, 1,934 participants who were missing information on serum cotinine or self-reported smoking variables, and 1,853 participants who were missing other variables of interest such as sociodemographic and cardiovascular risk factors. The final sample size was 8,989 participants. These participants had similar sociodemographic characteristics compared with the overall NHANES 1999–2004 population. The NCHS Research Ethics Review Board approved the NHANES protocols. All the participants provided oral and written informed consent.

*Blood and urine cadmium.* Blood and urine cadmium were measured at the Environmental Health Laboratory (Atlanta, GA, USA), Centers for Disease Control and Prevention, National Center for Environmental Health (NCEH). Extensive quality control procedures were followed including confirmation that collection and storage materials were not contaminated with background cadmium or other metals (NCHS 2008).

Blood cadmium and lead were measured simultaneously in whole blood using a multielement atomic absorption spectrometer with Zeeman background correction (SIMAA 6000 model; PerkinElmer, Norwalk, CT, USA) in 1999–2002 and an inductively coupled plasma-mass spectrometer (PerkinElmer/SCIEX model 500; PerkinElmer, Shelton, CT, USA) in 2003–2004. National Institute of Standards and Technology whole blood standard reference materials were used for external calibration (NCHS 2008). The interassay coefficients of variation for blood cadmium ranged from 3.2% to 9.4%. The limit of detection (LOD) was 0.3 μg/L in NHANES 1999–2002 and 0.2 μg/L in NHANES 2003–2004, resulting in 15% and 5% of observations below the LOD, respectively.

Urine cadmium was measured only in a randomized subsample of one-third of the 1999–2004 NHANES population (*n* = 2,867 in our study population). Thus, measurements of urine cadmium were missing completely at random in the remaining two-thirds of the study population. Urine cadmium was measured using inductively coupled plasma-mass spectrometry (PerkinElmer/SCIEX model 500). The interassay coefficients of variation for urine cadmium ranged from 1.2% to 4.7%. The LOD was 0.06 μg/L, which resulted in 3% of the observations falling below the LOD. In NHANES 1999–2002, cadmium levels in urine were corrected for interference from molybdenum oxide. NIST urine reference materials were used for external calibration (NCHS 2008). Urine cadmium data was reported in micrograms cadmium per gram creatinine. Urine creatinine was measured by the modified kinetic Jaffé method (NCHS 2008).

For participants with blood cadmium concentrations below the LOD, and for participants with urine cadmium concentrations missing completely at random, cadmium concentrations were imputed as the median of the subject-specific posterior distribution of predicted levels obtained from a Markov Chain Monte Carlo with Gibbs sampling nested linear model (Tellez-Plaza et al. 2010). The variables used to predict cadmium levels were selected by a backward stepwise process using linear regression models that included age, sex, smoking status, serum cotinine, and blood or urine cadmium. The imputation methods have been described in detail elsewhere (Tellez-Plaza et al. 2010). Urine cadmium measurements below the LOD were imputed as the LOD divided by the square root of two (NCHS 2008).

*Baseline data collection.* Information on age; sex; race/ethnicity; education; menopause status; smoking; and medication for treating hypertension, diabetes, and hypercholesterolemia was based on self-reported information. Body mass index and blood pressure were measured during the examination (NCHS 2008). Hypertension was defined as a mean systolic blood pressure ≥ 140 mmHg, a mean diastolic blood pressure ≥ 90 mmHg, a self-reported physician diagnosis, or medication use. Diabetes was defined as a fasting glucose ≥ 126 mg/dL, a nonfasting glucose ≥ 200 mg/dL, a self-reported physician diagnosis, or medication use. Serum C-reactive protein was analyzed using high-sensitivity latex-enhanced nephelometry using the Behring Nephelometer Analyzer II (Dade-Behring Diagnostics Inc., Somerville, NJ, USA). Serum total cholesterol was measured enzymatically using the Cholesterol High Performance reagent (no. 704036; Roche Diagnostics, Indianapolis, IN, USA). High density-lipoprotein (HDL) cholesterol was measured using a direct HDL reagent (no. 1661442; Roche Diagnostics). High cholesterol was defined as a serum total cholesterol > 200 mg/dL or medication use. Serum cotinine was measured by an isotope-dilution high-performance liquid chromatography/atmospheric pressure chemical ionization tandem mass spectrometric method (NCHS 2008). Serum creatinine was measured using a kinetic rate Jaffé method in a Hitachi 704 multichannel analyzer (Boehringer Mannheim Diagnostics, Indianapolis, IN, USA). Estimated glomerular filtration rate (eGFR) was calculated from calibrated creatinine, age, sex, and race/ethnicity using the Modification of Diet in Renal Disease Study formula (Selvin et al. 2007).

*Mortality follow-up.* NHANES 1999–2004 participants were followed for mortality through 31 December 2006. Vital status and cause of death were determined by probabilistic matching between NHANES records and death certificates from the National Death Index (NDI) (NCHS 2010). The NCHS submitted NHANES records to the NDI for the implementation of a probabilistic algorithm that used identifying data elements such as social security number, first name, middle name, last name, date of birth, race, sex, state of birth, and state of residence to match records according to established criteria (NCHS 2010). Before the matching algorithm was applied, each record was screened to determine if it contained sufficient combinations of identifying data elements, with < 0.2% of NHANES population being considered ineligible for the matching. A calibration study on NHANES I Epidemiological Follow-up Study, which used similar methodology, found that 96.1% of deceased participants and 99.4% of living participants were correctly classified (NCHS 2010). The cause of death was determined using the underlying cause listed on death certificates, and was coded using the *International Classification of Diseases*, *10th Revision* (ICD-10; World Health Organization 2007). Cause-specific mortality was ascertained for CVD (ICD-10 codes I00-I78), heart disease (ICD-10 codes I00-I09, I11, I13, I20-I51) and ischemic heart disease (ICD-10 codes I20-I25). NCHS ensures that the identity of the participants is not disclosed (NCHS 2010). All direct identifiers, as well as any characteristics that might lead to identification, were omitted from the linked dataset used in the present study.

Follow-up time for each individual was calculated as the difference between the age at the date of the NHANES examination and the age at the date of death, age on 31 December 2006, or age 90 years, whichever occurred first. Follow-up was censored at age 90 years because of the high mortality after this age and the low number of participants who were contributing person-time experience.

*Statistical methods.* To perform all the statistical analyses we used the survey package (Lumley 2004) in R software (version 2.12.1; R Development Core Team 2011) to account for the complex sampling design and weights of the NHANES and to obtain appropriate standard errors. Blood and urine cadmium levels were right skewed and log-transformed for the analyses. Cut-offs for blood cadmium quintiles were based on weighted distributions in the study sample. Cut-offs for creatinine-corrected urine cadmium quantiles were based on weighted distributions in the originally measured one-third random subsample.

We estimated hazard ratios [95% confidence intervals (CIs)] for mortality end points using Cox proportional hazards regression with age as the time scale and individual starting follow-up times (age at examination) treated as the staggered entries. Cadmium was modeled as a log-linear term, which was used to estimate hazard ratios for mortality comparing the 80th with the 20th percentiles of the blood and creatinine-corrected urine cadmium distributions. To test for nonlinear relationships in all-cause and CVD mortality models, we used restricted quadratic splines with knots at the 20th, 50th, and 80th percentiles of each cadmium distribution, and we applied the Wald test adjusted for the survey design to the nonlinear spline terms.We observed no statistically significant departures from linearity (*p*-values for nonlinearity in all-cause and CVD mortality models were 0.40 and 0.20, respectively, for blood cadmium and 0.56 and 0.89, respectively, for urine cadmium). The level of statistical significance used for hypothesis testing was 0.05.

Statistical models were progressively adjusted to evaluate the potential confounding effect of different groups of variables. Model 1 accounted for sociodemographic variables including race/ethnicity (non-Hispanic white, non-Hispanic black, Mexican-American, other), sex (men, women), education (≥ high school, < high school) and low income (≥ $20,000, < $20,000). Model 2 further adjusted for established CVD risk factors including postmenopausal status for women (no, yes), body mass index (continuous, kilograms per meter squared), hypertension (no, yes), diabetes (no, yes), blood lead (continuous, log micrograms per deciliter), total cholesterol (continuous, milligrams per deciliter), HDL cholesterol (continuous, milligrams per deciliter), cholesterol lowering medication (no, yes), C-reactive protein (continuous, log milligrams per liter), and estimated glomerular filtration rate (eGFR; continuous, milliliter per minute per 1.73 meters squared). Smoking could be a major confounder. Thus, in model 3, we further adjusted for current smoking status (never, former, current), intensity of current exposure to tobacco smoke using serum cotinine levels (continuous, log nanograms per milliliter), and cumulative smoking using number of pack-years (modeled as restricted cubic splines with knots at 10, 20, and 30 pack-years). The assumption of hazards proportionality was assessed visually based on the smoothed relationship between time and scaled Schoenfeld residuals (Grambsch and Therneau 1994). We observed no major departures from proportionality. The primary statistical analyses were conducted in the dataset including imputed cadmium values. As sensitivity analyses, we repeated the analyses restricting the sample to the one-third random subsample with the originally measured urine cadmium levels.

We had *a priori* interest on evaluating differential associations by sex and smoking subgroups. Subgroup analyses were conducted by including interaction terms for log-transformed blood or creatinine-corrected urine cadmium with the corresponding indicator variables for subgroups defined by sex (men, women) and smoking status (never, former, current) in separate models. In the Cox models, the nonparametric baseline hazards were allowed to differ by subgroup categories. We used the Wald test that was adjusted for the survey design to obtain the *p*-values for the interaction.

Adjusted population attributable risks (PARs) for cadmium exposure were calculated by adapting the standard formula PAR = 1 – Σ*_j_*Σ*_i_ p_ij_*/*RR_i_*_|_*_j_* (Benichou 2001; Bruzzi et al. 1985) to the survey data. In this formula, the subscript *i* denotes one of two categories of cadmium exposure (with each participant classified as exposed or unexposed if above or below the percentile being used to calculate the PAR, respectively), the subscript *j* is an index for all strata obtained after cross-classifying the study sample for all adjusted covariates, *p_ij_* is the survey-weighted proportion of cases over all cases in the study population in each stratum after cross-classifying the dichotomous cadmium exposure and all adjusted covariates, and *RR_i_*_|_*_j_* is the adjusted hazard ratio for mortality comparing participants exposed to cadmium with those unexposed in stratum *j* of covariates. We calculated adjusted PARs for each percentile of cadmium exposure starting at the 10th percentile of each cadmium distribution and displayed the results after smoothing using lowess with 0.90 bandwidth. For each cadmium concentration cut-off value, adjusted PARs thus represent the estimated fraction of deaths that would be avoided in the population had cadmium exposure in participants with levels above that concentration been similar to cadmium exposure in participants with levels below that concentration, assuming that the effects of cadmium are causal and that other risk factors remained unchanged.

## Results

The geometric means of blood and urine cadmium were 0.44 μg/L and 0.28 μg/g creatinine, respectively. The corresponding geometric means for men and for women were, respectively, 0.41 and 0.46 μg/L for blood cadmium and 0.22 and 0.34 μg/g creatinine for urine cadmium. The Spearman correlation between blood and urine cadmium was 0.61. Blood and urine cadmium were positively associated with age, postmenopausal status, current smoking, blood lead, hypertension, total cholesterol, and less than a high school education; they were inversely associated with eGFR (Table 1). Compared with urine cadmium, blood cadmium was more strongly related to current smoking status and serum cotinine concentrations, and less strongly related to former smoking status. Blood and urine cadmium concentrations were similarly related to cumulative smoking measured as pack-years.

**Table 1:** Baseline characteristics of study participants by cadmium tertiles.

Blood cadmium (μg/L)				Urine cadmium (μg/g creatinine)			
Characteristic	< 0.3	0.3–0.5	≥ 0.5	p-Trenda	< 0.20	0.20–0.41	≥ 0.41	p-Trenda
Age (years; mean ± SE)		42.4 ± 0.4	51.1 ± 0.4	49.1 ± 0.4	< 0.001		35.0 ± 0.4	47.6 ± 0.4	58.1 ± 0.4	< 0.001
Men (% ± SE)		55.6 ± 1.0	41.0 ± 1.2	46.0 ± 1.0	< 0.001		67.2 ± 1.1	45.7 ± 0.9	32.3 ± 0.9	< 0.001
Postmenopausal womenb (% ± SE)		33.4 ± 1.3	57.4 ± 1.5	55.7 ± 1.8	< 0.001		11.9 ± 1.4	41.9 ± 1.3	73.4 ± 1.6	< 0.001
Non-Hispanic white (% ± SE)		74.3 ± 1.7	72.4 ± 2.0	75.5 ± 2.0	0.31		70.7 ± 1.6	73.3 ± 1.9	79.2 ± 1.9	< 0.001
Non-Hispanic black (% ± SE)		10.0 ± 0.9	7.6 ± 1.0	9.8 ± 1.1	0.58		10.8 ± 1.0	9.6 ± 1.0	7.3 ± 0.8	< 0.001
Mexican-American (% ± SE)		7.5 ± 1.0	7.9 ± 1.2	4.7 ± 0.8	< 0.001		8.9 ± 1.0	6.9 ± 1.0	4.2 ± 0.7	< 0.001
< High school (% ± SE)		12.6 ± 0.8	19.7 ± 1.1	27.1 ± 0.9	< 0.001		12.0 ± 0.7	18.8 ± 1.1	27.2 ± 1.2	< 0.001
Annual household income < $20,000 (% ± SE)		17.3 ± 0.8	21.6 ± 1.1	31.6 ± 1.6	< 0.001		18.4 ± 1.0	20.6 ± 1.1	31.1 ± 1.6	< 0.001
BMI (kg/m2; mean ± SE)		28.4 ± 0.2	28.2 ± 0.2	27.3 ± 0.1	< 0.001		28.1 ± 0.2	28.1 ± 0.1	27.8 ± 0.2	0.06
Never-smoker (% ± SE)		78.2 ± 0.8	59.5 ± 1.4	17.9 ± 0.9	< 0.001		79.9 ± 0.9	51.9 ± 1.1	28.5 ± 1.1	< 0.001
Former smoker (% ± SE)		19.4 ± 0.8	31.3 ± 1.4	17.5 ± 0.8	0.001		11.2 ± 0.8	24.6 ± 1.2	30.2 ± 1.1	< 0.001
Current smoker (% ± SE)		2.4 ± 0.3	9.2 ± 0.7	64.6 ± 1.3	< 0.001		8.9 ± 0.7	23.5 ± 1.3	41.4 ± 1.5	< 0.001
Serum cotinine [ng/mL; GM (95% CI)]		0.07 (0.06, 0.08)	0.11 (0.10, 0.13)	11.46 (9.08, 14.46)	< 0.001		0.12 (0.10, 0.15)	0.35 (0.27, 0.46)	1.66 (1.26, 2.18)	< 0.001
Cumulative smoking [pack-years; mean (95% CI)]		2.8 (0.2)	8.0 (0.5)	22.9 (0.7)	< 0.001		1.5 (0.1)	8.2 (0.3)	23.4 (0.7)	< 0.001
C-reactive protein [mg/L; GM (95% CI)]		0.16 (0.15, 0.17)	0.20 (0.19, 0.21)	0.22 (0.21, 0.24)	< 0.001		0.14 (0.13, 0.15)	0.19 (0.18, 0.20)	0.26 (0.25, 0.27)	< 0.001
Hypertension (% ± SE)		30.8 ± 1.1	40.3 ± 1.1	38.4 ± 1.3	< 0.001		22.3 ± 1.2	36.0 ± 1.0	50.0 ± 1.2	< 0.001
Diabetes (% ± SE)		7.4 ± 0.6	8.4 ± 0.6	7.9 ± 0.5	0.64		4.3 ± 0.4	7.8 ± 0.6	11.5 ± 0.8	< 0.001
eGFR < 60 mL/min/1.73m2 (% ± SE)		5.1 ± 0.5	9.8 ± 0.7	10.7 ± 0.7	< 0.001		2.7 ± 0.4	7.8 ± 0.7	14.4 ± 0.8	< 0.001
Total cholesterol (mg/dL; mean ± SE)		199.8 ± 1.0	204.9 ± 1.1	205.9 ± 1.1	< 0.001		194.9 ± 1.0	203.0 ± 1.0	212.1 ± 1.1	< 0.001
HDL-cholesterol (mg/dL; mean ± SE)		51.5 ± 0.4	53.2 ± 0.4	51.6 ± 0.5	0.50		49.9 ± 0.4	52.4 ± 0.5	53.8 ± 0.4	< 0.001
Cholesterol-lowering medication (% ± SE)		9.7 ± 0.5	12.8 ± 0.9	11.7 ± 0.8	0.10		4.3 ± 0.5	11.2 ± 0.7	18.4 ± 0.8	< 0.001
Blood lead [μg/dL; GM (95% CI)]		1.29 (1.24, 1.34)	1.63 (1.57, 1.69)	2.11 (2.03, 2.20)	< 0.001		1.30 (1.24, 1.36)	1.60 (1.54, 1.66)	2.03 (1.95, 2.10)	< 0.001
Abbreviations: BMI, body mass index; GM, geometric mean. To convert blood cadmium from micrograms per liter to nanomoles per liter, multiply by 8.897. To convert urine cadmium from micrograms per gram creatinine to nanomoles per millimole creatinine, multiply by 1.006. ap-Values for trend were obtained by introducing the median cadmium level for each tertile as a continuous variable in the regression model. bSubsample of women (n = 4,497).										

**Figure 1:**
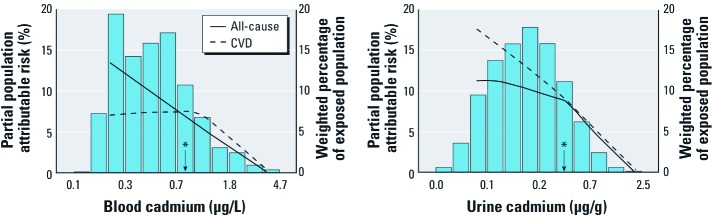
Partial PAR associated with cadmium exposure. Relative risks for calculating the partial PAR were obtained from fully adjusted Cox proportional hazards models (model 3). The partial PAR represents the estimated fraction of deaths that would be avoided in the population had cadmium exposure in participants with levels above a given percentile of the cadmium distribution been similar to cadmium exposure in participants with levels below that concentration, assuming that the effects of cadmium are causal and that other risk factors remained unchanged. The asterisk indicates the 80th percentiles of blood and urine cadmium distributions (0.80 μg/L and 0.57 μg/g creatinine, respectively). Bars indicate the weighted percent of the exposure in the population and lines indicate estimated PARs according to blood or urine cadmium concentrations. For example, model estimates suggest that if cadmium exposure in participants with urine cadmium concentrations above the 80th percentile of the distribution was reduced to that of participants below the 80th percentile, 9.2% of cardiovascular deaths in the U.S. population would be avoided.

The mean follow-up time was 4.8 years for participants who were alive at the end of follow-up, and 3.2 years for participants who died before the end of follow-up. The numbers of deaths due to all causes, CVD, heart disease, and ischemic heart disease were 524, 191, 113, and 88, respectively. The fully adjusted hazard ratios (95% CIs) that compared the 80th percentile with the 20th percentiles of the blood and urine cadmium distributions were 1.50 (95% CI: 1.07, 2.10) and 1.52 (95% CI: 1.00, 2.29), respectively, for all-cause mortality, 1.69 (95% CI: 1.03, 2.77) and 1.74 (95% CI: 1.07, 2.83) for CVD mortality, 1.98 (95% CI: 1.11, 3.54) and 2.53 (95% CI: 1.54, 4.16) for heart disease mortality and 1.73 (95% CI: 0.88, 3.40) and 2.09 (95% CI: 1.06, 4.13) for coronary heart disease mortality (Table 2). The partial PARs associated with having cadmium concentrations above the 80th percentile of the blood (0.80 μg/ L) and urine (0.57 μg/g) cadmium distributions were 7.0 and 8.8%, respectively, for all cause mortality and 7.5 and 9.2%, respectively, for CVD mortality (Figure 1).

**Table 2:** Hazard ratio (HR) of mortality end points comparing the 80th with the 20th percentiles of cadmium distributions [HR (95% CI)].

Blood cadmium (μg/L)					Urine cadmium (μg/g)				
Mortality (no. of deaths)	Model 1^a^	Model 2^b^	Model 3^c^	Model 1^a^	Model 2^b^	Model 3^c^
All-cause (n = 524)		2.20 (1.57, 3.09)		2.11 (1.54, 2.90)		1.50 (1.07, 2.10)		2.33 (1.72, 3.15)		2.27 (1.70, 3.04)		1.52 (1.00, 2.29)
Cardiovascular disease (n = 191)		2.37 (1.47, 3.81)		2.32 (1.41, 3.82)		1.69 (1.03, 2.77)		2.32 (1.61, 3.35)		2.41 (1.64, 3.55)		1.74 (1.07, 2.83)
Heart disease (n = 113)		2.72 (1.45, 5.08)		2.95 (1.57, 5.54)		1.98 (1.11, 3.54)		2.93 (1.91, 4.50)		3.34 (2.18, 5.13)		2.53 (1.54, 4.16)
Ischemic heart disease (n = 88)		2.21 (1.17, 4.16)		2.37 (1.20, 4.68)		1.73 (0.88, 3.40)		2.52 (1.54, 4.11)		2.85 (1.76, 4.61)		2.09 (1.06, 4.13)
The 80th and 20th percentiles were 0.80 μg/L and 0.22 μg/L, respectively, for blood cadmium and 0.57 μg/g and 0.14 μg/g, respectively, for urine cadmium. To convert blood cadmium from micrograms per liter to nanomole per liter, multiply by 8.897. To convert urine cadmium from microgram per gram creatinine to nanomole per millimole creatinine, multiply by 1.006. aModel 1 adjusted for sex (men, women), education (≥ high school, < high school), annual household income (≥ $20,000, < $20,000) and race/ethnicity (non-Hispanic white, non-Hispanic black, Mexican-American, other). bModel 2 was model 1 with further adjustments for postmenopausal status for women (no, yes), body mass index (kg/m2), blood lead (log micrograms per deciliter), C-reactive protein (log milligrams per liter), total cholesterol (milligrams per deciliter), HDL cholesterol (milligrams per deciliter), cholesterol lowering medication use (no, yes), hypertension (no, yes), diabetes (no, yes), estimated glomerular filtration rate (milliliter per minute per 1.73 meters squared). cModel 3 was model 2 with further adjustments for smoking status (never, former, current), cumulative smoking dose (modeled as restricted cubic splines with knots at 10, 20, and 30 pack-years) and serum cotinine (log nanogram per milliliter).												

In the subgroup analysis, the association of blood and urine cadmium with all-cause and CVD mortality was evident in most subgroups defined by sex and smoking status [see Supplemental Material, Tables 1 and 2 (http://dx.doi.org/10.1289/ehp.1104352)]. Current smokers, however, had a higher risk of CVD mortality with increasing blood and urine cadmium concentrations (see Supplemental Material, Table 2). Among never-smokers, the hazard ratios for cardiovascular mortality were 1.17 (95% CI: 0.53, 2.55) and 1.98 (95% CI: 0.90, 4.35) for blood and urine cadmium, respectively (see Supplemental Material, Table 2). In the sensitivity analyses that were performed in the one-third subset of participants with the originally measured urine cadmium, the hazard ratio point estimates for the 80th versus the 20th percentiles of cadmium were consistent overall (see Supplemental Material, Table 3). In the corresponding subgroup analysis, the estimates were also consistent with the results from the imputed dataset (see Supplemental Material, Table 4).

## Discussion

Cadmium exposure, as measured in blood and urine, was prospectively associated with all-cause and CVD mortality in a recent representative sample of U.S. adults after adjusting for sociodemographic and CVD risk factors, including smoking status, recent smoking dose, and cumulative smoking dose. Estimated PARs based on urine cadmium, the most reliable biomarker of cumulative exposure, suggest that increasing cadmium exposure is associated with a progressive increase in avoidable cardiovascular deaths. These findings are remarkable considering the relatively low levels of exposure in the U.S. general population.

Cadmium is absorbed through the respiratory and digestive tracts (Nordberg et al. 2007). After absorption, cadmium progressively accumulates in the kidney (half of body burden), liver, pancreas, and the central nervous system (Nordberg et al. 2007). In urine, cadmium reflects kidney cadmium concentrations with a half-life of 15–30 years. Urine cadmium is considered a marker of cumulative body burden. In blood, cadmium concentrations are more dependent on daily exposure fluctuations, and blood cadmium is considered a marker of ongoing exposure (ATSDR 2008; Nordberg et al. 2007). In cadmium-exposed workers, however, blood cadmium showed a fast component with a half-life of 3–4 months and a slow component with a half-life of 10 years (Järup et al. 1983)—these findings suggest that blood cadmium also reflects body burden. In our study, blood cadmium was strongly associated with current smoking and with serum cotinine, a metabolite of nicotine that has a half-life of 16 hr (Benowitz et al. 2009). Urine cadmium, on the other hand, was more strongly associated with former-smoker status compared with blood cadmium.

Importantly, both blood and urine cadmium concentrations were similarly related to cumulative smoking measured as pack-years, which indicates that both can reflect cumulative exposure. Because blood cadmium can undergo short-term fluctuations from recent exposure, this fluctuation could translate into a nondifferential measurement error, which would bias the estimates toward the null in blood cadmium models. Indeed, this measurement error is supported by our data, because both biomarkers were associated with all-cause and CVD mortality, but associations were stronger for urine cadmium than for blood cadmium.

Prospective studies of cadmium levels and mortality are scarce and most have investigated mortality in populations exposed to relatively high cadmium concentrations (Nakagawa et al. 2006; Nawrot et al. 2008). In a study of a Belgium population (*n* = ~ 950), urine cadmium concentrations at baseline (geometric mean of 0.98 μg/24hr) were associated with all-cause mortality after almost 20 years of follow-up (Nawrot et al. 2008). In a study conducted in Japan’s Kakeshi river basin (*n* = 3,119), urine cadmium concentrations at baseline (geometric mean of 5.99 μg/g) were also associated with all-cause mortality after 15 years of follow-up (Nakagawa et al. 2006). In 1988–1994 NHANES, urine cadmium was associated with mortality among men but not among women (geometric means of 0.28 μg/g and 0.40 μg/g for men and women, respectively) (Menke et al. 2009). At substantially lower concentrations of exposure in the more recent 1999–2004 NHANES cohort, we found no evidence of effect modification by sex. It is possible that the sex differences are present only at higher concentrations of exposure, but they also could be due to random sampling variability. It will be necessary to reproduce the findings in other general populations with similarly low concentrations of exposure to cadmium.

In our data, we found a stronger association between cadmium exposure and cardiovascular mortality end points compared to the association with all-cause mortality. These findings are consistent with dose–response relationships in the previous 1988–1994 NHANES prospective study, and also with cross-sectional associations observed between blood and urine cadmium with peripheral arterial disease (Navas-Acien et al. 2004, 2005; Tellez-Plaza et al. 2010), increased intima-media thickness (Messner et al. 2009), electrocardiographically assessed myocardial infarction (Everett and Frithsen 2008), and self-reported heart failure and stroke (Lee et al. 2011; Peters et al. 2010). In our study, we could not evaluate the specific associations with stroke or heart failure, because the number of deaths for those causes was very small. Prospective studies in populations exposed to higher cadmium concentrations from Japan (Nakagawa et al. 2006) and the United States (Menke et al. 2009) also reported associations of cadmium with cardiovascular mortality end points. In a study in Belgium, however, Nawrot et al. (2008) observed that the prospective association of cadmium exposure with CVD mortality was borderline statistically significant, which was likely due to the relatively small number of cardiovascular events in their study population.

A potential effect of cadmium on CVD is supported by experimental and mechanistic evidence that has shown that cadmium causes endothelial cell dysfunction *in vitro* and accelerates atherosclerotic plaque formation *in vivo* (Messner et al. 2009). Several mechanisms may explain the role of cadmium in promoting atherosclerosis. In some experimental studies, cadmium contributes to the formation of reactive oxygen species (Prozialeck et al. 2008; Valko et al. 2005) and interferes with anti-oxidative stress responses (Bell and Vallee 2009; Jin et al. 1998; Maret 2009; Valko et al. 2005). Cadmium also inhibits endothelial cell cycle through the induction of an atypical form of apoptosis, which involves attraction and activation of macrophages (Messner et al. 2009). Other mechanisms, such as cadmium-related hypertension (Bilgen et al. 2003; Satarug et al. 2006; Varoni et al. 2003), estrogenic activity (Stoica et al. 2000), and epigenetic changes (Jiang et al. 2008; Takiguchi et al. 2003) could also contribute to the cardiovascular effects of cadmium.

In addition to a relatively short follow-up, which limited our ability to evaluate dose–response relationships in cause-specific end points, our study has other limitations. First, we had only mortality outcomes obtained from death certificates, which is limited because the underlying cause of death can be miscoded. We also did not have incident morbidity events, and, as a consequence, our study can provide only a limited view of all potential cardiovascular health effects of cadmium. Studies using medical records are needed, including not only fatal but also nonfatal incident events. Second, because of missing values for variables that were needed for adjustment, we excluded a number of participants from the analytical sample. However, participants in the study population were comparable to overall NHANES 1999–2004 population with respect to sociodemographic characteristics. Third, we measured cadmium biomarkers at only one point in time, and underestimation of the associations because of nondifferential measurement error is possible. Fourth, a substantial proportion of study participants had blood cadmium levels below the LOD, and two-thirds of the study participants who were not randomly sampled for cadmium measurements had urine cadmium levels missing completely at random. We attempted to overcome this limitation by using a prediction model to impute the missing cadmium data (Tellez-Plaza et al. 2010). Fifth, we adjusted our models for sociodemographic and cardiovascular risk factors using a large number of variables. Although this adjustment using a large number of variable may have resulted in overadjustment, adjusting for both sociodemographic and CVD risk factors was considered important to minimize residual confounding. Finally, residual confounding by smoking is a concern because tobacco is a major source of exposure to cadmium in the general population (ATSDR 2008; Nordberg et al. 2007) as well as an established cardiovascular risk factor (Department of Health and Human Services 2010; Roger et al. 2011). In subgroup analyses by smoking, the association was stronger among current smokers for both blood and urine cadmium, although the interaction was only statistically significant for blood cadmium. Potentially, cadmium could have synergistic effects with other toxicants in tobacco. Remarkably, urine cadmium was associated with CVD mortality among never smokers—a finding that supports that cumulative cadmium exposure is a CVD risk factor that is independent of tobacco smoking. In addition, the associations between cadmium and mortality end points remained after extensive adjustment for smoking status, recent smoking dose, and cumulative smoking dose, making residual confounding less likely.

Strengths of our study include the sampling design that allows our findings to be generalized to the U.S. general population, the availability of detailed information for adjusting multiple risk factors, including serum cotinine, and the high quality of NHANES procedures, including laboratory methods for cadmium exposure and methods for matching NHANES participants with the National Death Index.

Our findings strongly suggest that cadmium, at low levels of exposure, remains a determinant of all-cause and CVD mortality. It will be important to reproduce these findings in other populations exposed to similarly low cadmium concentrations, with longer follow-up periods and with incident events. Although cadmium exposure has declined substantially in the United States during the past few decades (Tellez-Plaza et al. 2012), additional public health efforts, for instance reinforcing tobacco control; reducing cadmium in air, soils, and food; and evaluating the environmental impact of nonrecycled cadmium-containing products and cadmium-containing fertilizers, may contribute to the reduction of cadmium-related disease burden in future generations.

## Supplemental Material

(168 KB) PDF
