# Supplementary material for: Cadmium Exposure and All-Cause and Cardiovascular Mortality in the U.S. General Population
Source: Environ Health Perspect. 2012 Apr 2;120(7):1017–22. doi: 10.1289/ehp.1104352 (PMC3404657; doi:10.1289/ehp.1104352)
Supplement: (168 KB) PDF [file ehp.1104352.s001.pdf]

Supplemental Material

Title: Cadmium Exposure and All Cause and Cardiovascular Mortality in the US General Population

Authors: Maria Tellez-Plaza, Ana Navas-Acien, Andy Menke, Ciprian M. Crainiceanu, Roberto Pastor-Barriuso and Eliseo Guallar.

TABLE OF CONTENTS

Supplemental Material, Table 1..page 2

Supplemental Material, Table 2..page 3

Supplemental Material, Table 3..page 4

Supplemental Material, Table 4..page 5

Supplemental Material, Table 1. Hazard ratio of all cause mortality comparing the 80th to the 20th percentiles of cadmium distributions by sex and smoking status.

			Blood Cadmium, µg/L		***Urine Cadmium, µg/g***	
**Subgroups**	**Cases**	**Non cases**	**HR (95% CI)**	**P-int**	**HR (95% CI)**	**P-int**
Sex						
Men	303	4,189	1.55 (1.11, 2.16)	0.71	1.78 (1.09, 2.90)	0.19
Women	221	4,276	1.41 (0.87, 2.30)		1.27 (0.76, 2.11)	
Smoking						
Never	205	4,698	1.19 (0.70, 2.02)	0.20	1.35 (0.79, 2.32)	0.47
Former	202	1,956	1.62 (0.88, 2.98)		1.77 (0.93, 3.35)	
Current	117	1,811	2.21 (1.32, 3.70)		2.07 (1.08, 3.97)	
Overall	524	8,465	1.50 (1.07, 2.10)		1.52 (1.00, 2.29)	

Abbreviations: CI, confidence interval; HR, hazard ratio; and P-int, P-interaction.

The 80th and 20th percentiles were 0.80 µg/L and 0.22 µg/L, respectively, for blood cadmium and 0.57 µg/g and 0.14 µg/g, respectively, for urine cadmium. To convert blood cadmium from µg/L to nmol/L, multiply by 8.897. To convert urine cadmium from µg/g creatinine to nmol/mmol creatinine, multiply by 1.006. Analyses were conducted using Cox models with interaction terms for log-transformed cadmium with the corresponding indicator variables for subgroups. In the Cox models, the nonparametric baseline hazards were allowed to differ by subgroup categories. P-values for the interaction were obtained by using the Wald test adjusted for the survey design. Model adjusted for sex (men, women), race-ethnicity (non-Hispanic White, non-Hispanic Black, Mexican-American, other), education (≥ high school, < high school), annual household income (≥$20,000, < $20,000), post-menopausal status for women (no, yes), body mass index (kg/m^2), blood lead (log µg/dL), C-reactive protein (log mg/L), total cholesterol (mg/dL), HDL cholesterol (mg/dL), cholesterol lowering medication use (no, yes), hypertension (no, yes), diabetes (no, yes), estimated glomerular filtration rate (ml/min/1.73m^2), pack-years (restricted cubic splines with knots at 10, 20 and 30 pack-years), smoking (never, former, current), and serum cotinine (log ng/mL).

Supplemental Material, Table 2. Hazard ratio of cardiovascular mortality comparing the 80th to the 20th percentiles of cadmium distributions by sex and smoking status.

			Blood Cadmium, µg/L		***Urine Cadmium, µg/g***	
**Subgroups**	**Cases**	**Non cases**	**HR (95% CI)**	**P-int**	**HR (95% CI)**	**P-int**
Sex						
Men	106	4,386	1.50 (0.84, 2.68)	0.53	1.87 (0.96, 3.66)	0.74
Women	81	4,416	1.90 (0.97, 3.71)		1.62 (0.87, 3.01)	
Smoking						
Never	77	4,826	1.17 (0.53, 2.55)	0.01	1.98 (0.90, 4.35)	0.08
Former	71	2,087	1.22 (0.47, 3.16)		0.92 (0.33, 2.59)	
Current	39	1,889	4.36 (2.28, 8.36)		3.99 (2.02, 7.86)	
Overall	187	8,802	1.69 (1.03, 2.77)		1.74 (1.07, 2.83)	

Abbreviations: CI, confidence interval; HR, hazard ratio; and P-int, P-interaction.

The 80th and 20th percentiles were 0.80 µg/L and 0.22 µg/L, respectively, for blood cadmium and 0.57 µg/g and 0.14 µg/g, respectively, for urine cadmium. To convert blood cadmium from µg/L to nmol/L, multiply by 8.897. To convert urine cadmium from µg/g creatinine to nmol/mmol creatinine, multiply by 1.006. Analyses were conducted using Cox models with interaction terms for log-transformed cadmium with the corresponding indicator variables for subgroups. In the Cox models, the nonparametric baseline hazards were allowed to differ by subgroup categories. P-values for the interaction were obtained by using the Wald test adjusted for the survey design. Model adjusted for sex (men, women), race-ethnicity (non-Hispanic White, non-Hispanic Black, Mexican-American, other), education (≥ high school, < high school), annual household income (≥$20,000, < $20,000), post-menopausal status for women (no, yes), body mass index (kg/m2) , blood lead (log µg/dL), C-reactive protein (log mg/L), total cholesterol (mg/dL), HDL cholesterol (mg/dL), cholesterol lowering medication use (no, yes), hypertension (no, yes), diabetes (no, yes), estimated glomerular filtration rate (ml/min/1.73m2), pack-years (restricted cubic splines with knots at 10, 20 and 30 pack-years), smoking (never, former, current), and serum cotinine (log ng/mL).

Supplemental Material, Table 3. Hazard ratio of mortality endpoints comparing the 80th to the 20th percentiles of cadmium distributions in a 1/3 random subsample (N=2,867)

	Blood Cadmium, μg/L			*Urine Cadmium, μg/g*		
	**Model 1[a]**	**Model 2[b]**	**Model 3[c]**	**Model 1[a]**	**Model 2[b]**	**Model 3[c]**
**Mortality (number of cases)**	**HR (95% CI)**	**HR (95% CI)**	**HR (95% CI)**	**HR (95% CI)**	**HR (95% CI)**	**HR (95% CI)**
All-cause (169)	2.19 (1.27, 3.77)	2.04 (1.24, 3.35)	1.96 (1.28, 2.99)	2.05 (1.40, 3.00)	2.01 (1.38, 2.94)	1.85 (1.26, 2.71)
Cardiovascular disease (60)	1.87 (0.78, 4.51)	1.77 (0.75, 4.20)	1.79 (0.90, 3.55)	2.07 (1.14, 3.76)	1.97 (1.04, 3.75)	1.77 (0.81, 3.87)
Heart disease (41)	2.03 (0.57, 7.23)	1.86 (0.66, 5.23)	1.66 (0.85, 3.27)	3.71 (2.04, 6.75)	3.75 (1.67, 8.41)	3.63 (1.22, 10.87)
Ischemic heart disease (32)	1.67 (0.35, 7.85)	1.57 (0.57, 4.33)*	1.83 (0.79, 4.23)*	3.34 (1.48, 7.55)	3.03 (1.25, 7.31)*	3.21 (0.99, 10.44)*

Abbreviations: CI, confidence interval; and HR, hazard ratio.

The 80th and 20th percentiles were 0.80 μg/L and 0.22 μg/L, respectively, for blood cadmium and 0.57 μg/g and 0.14 μg/g, respectively, for urine cadmium.

[a]Model 1 adjusted for sex (men, women), education (≥ high school, < high school), annual household income (≥$20,000, < $20,000) and race-ethnicity (non-Hispanic White, non-Hispanic Black, Mexican-American, other).

[b]Model 2 was model 1 further adjusted for post-menopausal status for women (no, yes), body mass index (kg/m^2), blood lead (log μg/L), C-reactive protein (log mg/L), total cholesterol (mg/dL), HDL cholesterol (mg/dL), cholesterol lowering medication use (no, yes), hypertension (no, yes), diabetes (no, yes), estimated glomerular filtration rate (ml/min/1.73m^2).

[c]Model 3 was model 2 further adjusted for smoking status (never, former, current), cumulative smoking dose (modeled as restricted cubic splines with knots at 10, 20 and 30 pack-years) and serum cotinine (log ng/L) *Not adjusted for post-menopausal status to avoid system singularities in the models.

Supplemental Material, Table 4. Hazard ratio for all cause and cardiovascular mortality comparing the 80th to the 20th percentiles of urine cadmium distribution, by sex and smoking, in a 1/3 random subsample (N=2,867)

	All cause mortality				*Cardiovascular mortality*			
**Subgroups**	**Cases**	**Non cases**	**HR (95% CI)**	**P-int**	**Cases**	**Non cases**	**HR (95% CI)**	**P-int**
Sex								
Men	98	1,354	2.25 (1.19, 4.28)	0.26	36	1,416	1.71 (0.65, 4.49)	0.97
Women	71	1,344	1.56 (1.04, 2.34)		24	1,391	1.67 (0.77, 3.61)	
Smoking								
Never	72	1,478	1.29 (0.74, 2.24)	0.06	27	1,523	1.92 (0.90, 4.10)	0.81
Former	52	652	3.28 (1.79, 6.00)		21	688	1.37 (0.45, 4.24)	
Current	40	568	2.21 (0.58, 8.41)		12	596	2.85 (0.23, 35.56)	
Overall	169	2,698	1.85 (1.26, 2.71)				1.77 (0.81, 3.87)	

Abbreviations: CI, confidence interval; HR, hazard ratio; and P-int, P-interaction.

The 80th and 20th percentiles were 0.57 µg/g and 0.14 µg/g, respectively, for urine cadmium. To convert blood lead from µg/dL to µmol/L, multiply by 0.0483. To convert urine cadmium from µg/g creatinine to nmol/mmol creatinine, multiply by 1.006. Analyses were conducted using Cox models with interaction terms for log-transformed cadmium with the corresponding indicator variables for subgroups. In the Cox models, the nonparametric baseline hazards were allowed to differ by subgroup categories. P-values for the interaction were obtained by using the Wald test adjusted for the survey design. Model adjusted for sex (men, women), race-ethnicity (non-Hispanic White, non-Hispanic Black, Mexican-American, other), education (≥ high school, < high school), annual household income (≥$20,000, < $20,000), post-menopausal status for women (no, yes), body mass index (kg/m2) , blood lead (log µg/dL), C-reactive protein (log mg/L), total cholesterol (mg/dL), HDL cholesterol (mg/dL), cholesterol lowering medication use (no, yes), hypertension (no, yes), diabetes (no, yes), estimated glomerular filtration rate (ml/min/1.73m2), pack-years (restricted cubic splines with knots at 10, 20 and 30 pack-years), smoking (never, former, current), and serum cotinine (log ng/mL).
